# Extending classical nucleation theory to consider curvature and real-gas effects

**DOI:** 10.1016/j.ultsonch.2025.107649

**Published:** 2025-10-30

**Authors:** Mazyar Dawoodian, Ould el Moctar

**Affiliations:** Institute for Sustainable and Autonomous Maritime Systems, University of Duisburg-Essen, 47057 Duisburg, Germany

**Keywords:** Cavitation, Classical nucleation theory, Molecular dynamics

## Abstract

This paper introduces a Classical Nucleation Theory framework that explicitly incorporates curvature-dependent surface tension (Tolman correction) and real-gas behavior (Van der Waals correction) to predict cavitation inception at nanoscale gaseous nuclei. Validation is achieved through molecular dynamics simulations. The findings highlight the significant role of nanoscale gaseous nuclei in lowering the tensile strength required for cavitation initiation. The results show that our new CNT formulation predicts lower cavitation pressures than the Blake threshold, closely matching molecular dynamics simulations. The Tolman correction is most relevant for nuclei below about 10 nm, while for larger nuclei its effect becomes negligible and the model reduces to a Van der Waals–only description. Finally, our results illustrate that differences between cavitation pressures using the Van der Waals and ideal gas models are greatest for smaller nuclei and lower temperatures.

## Graphical abstract

Cross-sectional view of a stabilized bulk nanobubble.

**Figure:**
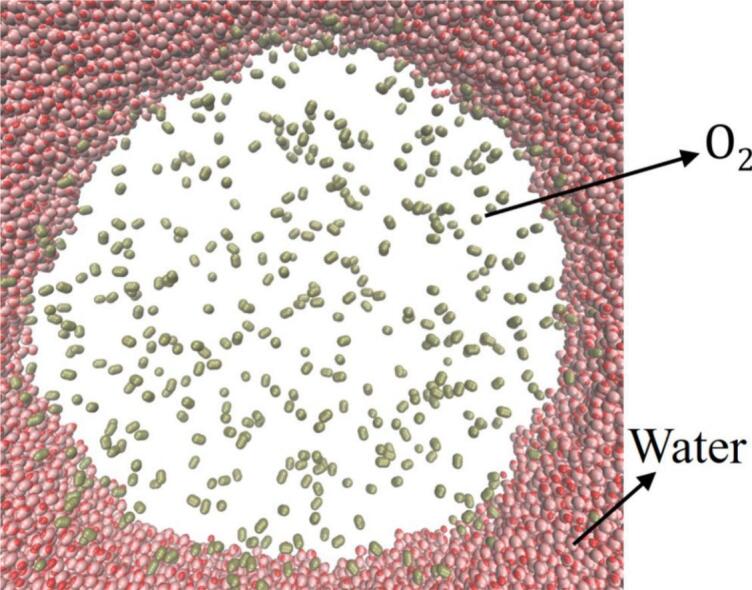

## 1 Introduction

Water’s strong cohesion enables it to withstand tension for prolonged durations [1,2]. Experimental measurements show that highly negative pressures can be sustained before bubble nucleation [3,4,5,6]. Recent research has focused on cavitation in water under tension due to its significance in various biological processes [7,8,9,10] and other industrial and medical applications [11,12,13], although its efficiency remains limited. Additionally, cavitation in water poses challenges in turbine and propeller design [14,15,16]. At high temperatures, different methods converge as the liquid cannot sustain significant tension. However, a much higher metastability level is achieved when examining cavitation in inclusions along an isochoric path [3,4,6] compared to other techniques [17,18] at low temperatures [19]. Critical cavitation pressure depends on factors like temperature, viscosity, surface tension, time, and strain rate [20,21], with surface tension and temperature being most influential [22].

Cavitation commonly initiates at pre-existing nuclei, a topic of extensive research [19,23,24,25,26]. The pioneering work of Harvey et al. [27] demonstrated the impact of pre-existing gas cavities on cavitation, attributing the measured low tensile strength on gaseous nuclei, a perspective subsequently supported by numerous studies. In purified and degassed liquids, with suppressed micrometre-sized nuclei [18], the measured tensile strengths (less than 30.00 MPa) remain notably lower than the theoretically expected 140.0 MPa [3,6], leaving the cause of this discrepancy unclear. Mørch [28] investigated the potential role of nanoscale gas bubbles/droplets as nuclei, which may be stabilized by the surface tension forces to achieve a gas diffusion balance with the adjacent liquid. Zhou et al. [29] further explored the stabilization of nanobubbles on a heterogeneous substrate through molecular dynamics simulations. Hong and Son [30] have demonstrated that bulk nanobubbles act as non-condensable gaseous nuclei, significantly lowering the acoustic cavitation threshold in water. However, the effects of such gaseous nanoscale nuclei on the cavitation dynamics remain poorly understood due to the challenging in observing them.

According to classical theories [31], nanobubbles are expected to dissolve rapidly due to the high Laplace pressure [32]. However, recent advancements show that they can survive for much longer periods, ranging from hours to even months [33]. For instance, Chen et al. [34] demonstrated that thermal capillary waves lower surface tension, counterbalancing the Laplace pressure and, thus, preventing rapid dissolution. By performing molecular dynamics simulations, they confirmed the stability of bulk nanobubbles even after 50 ns. Tan et al. [35] proposed electrostatic stabilization to predict equilibrium radii of 50–500 nm, which were consistent with experimental observations from dynamic light scattering and electron microscopy. Nirmalkar et al. [36] reported bulk nanobubbles (100–120 nm) lasting for months, stabilized by surface charge effects. Jadhav and Barigou [37] demonstrated stability in aqueous organic solvents for over three months due to gas oversaturation. Jin et al. [38] tracked microbubble shrinkage into stable nanobubbles (280 nm, zeta potential –33 mV), using dark-field microscopy, while [39] showed dynamic equilibrium under supersaturation. Additional support from [40,41,42,43] further validated the persistence of bulk nanobubbles, reinforcing their role as nucleation sites in our model. Gao et al. [44] employed molecular dynamics simulations to reveal that bulk nanobubble stabilization results from a combination of factors, including high internal gas density, electrostatic surface charges, and weakened interfacial hydrogen bonding. Ghaani et al. [45] demonstrated that external electric fields can massively enhance bulk nanobubble formation and stability. Fang et al. [46] showed that vibration can induce the formation of stable bulk nanobubbles in water, with sizes typically depending on vibration frequency and duration. Li et al. [47] demonstrated that nanoscale bulk gaseous nuclei play a crucial role in laser-induced cavitation, significantly influencing bubble dynamics.

Classical nucleation theory (CNT) is a foundational framework widely used to study homogeneous cavitation, with its predictive reliability validated by experiments [3] and molecular dynamics simulations [21]. CNT forms the foundation for modern nucleation models, including density functional theory [48] and kinetic nucleation theory [49]. These models have also been extended to heterogeneous cavitation, including cavitation on smooth, rigid surfaces [50]. However, Menzl et al. [21] revealed that CNT fails quantitatively at nanoscopic scales because it lacks critical microscopic information, including the curvature dependence of surface tension and thermal fluctuations that influence bubble expansion. Lohse and Prosperetti [51] emphasized the significance of these findings, noting that Menzl et al.’s work successfully bridges macroscopic and nanoscopic descriptions. They suggested that integrating molecular dynamics with continuum mechanics could enhance our understanding of fluid mechanics at small scales and improve broader predictive models. Recent studies by Gao et al. [52] and Alame and Mahesh [53] further illuminate the complexity of cavitation phenomena. Gao et al. [52] extended CNT for nanoscale nuclei and showed that nanoscale nuclei significantly reduce the tensile strength. Alame and Mahesh [53] proposed a Gibbs free energy framework linking homogeneous and heterogeneous nucleation, demonstrating gas content’s role in stabilizing nuclei and lowering energy barriers.

In addition, density functional theory (DFT) has been applied to cavitation inception and interfacial structure in water, resolving diffuse density profiles and free-energy barriers (Van der Waals–Cahn–Hilliard formulation) [54]. DFT naturally accounts for curvature dependence of surface tension and interfacial thickness, and has been extended to structured surfaces [55] and connected formally to CNT via squared-gradient approximations [56]. Recent DFT-type analyses of nanobubbles at interfaces [57] and MD–CNT ‘Seeding’ studies of cavitation rates [58] further delineate nanoscale behavior. However, DFT remains computationally demanding, generally restricted to homogeneous nucleation, and cannot be readily scaled to larger bubbles.

We present a CNT-based framework to predict cavitation inception pressure in nanoscale gaseous nuclei. The model incorporates Van der Waals corrections to capture intermolecular forces within confined gas pockets and the Tolman length to account for curvature-dependent surface tension effects (Section 2). Unlike previous CNT-based approaches, which typically assumed ideal gases or constant surface tension, our formulation combines real-gas and curvature corrections in a single framework tailored for gaseous nanonuclei. These refinements enable a more accurate assessment of cavitation induced by nanoscale gaseous nuclei. An additional advantage of our approach is its broad applicability: while molecular simulations are restricted to nuclei of only a few nanometers, the present formulation can be applied consistently across a wide range of bulk nanobubble sizes, from a few nanometers up to the micrometer scales. It is worth noting that for nuclei with $r_{0}<10$ nm, accounting for the Tolman correction is essential, while for larger nuclei its effect becomes negligible. By setting δ=0, the formulation effectively reduces to a Van der Waals–only description. In contrast to DFT-based approaches, our formulation retains the analytical tractability of CNT while incorporating essential nanoscale corrections explicitly for pre-existing gaseous nuclei. It thus provides a complementary continuum framework: whereas DFT excels in molecular-level fidelity for homogeneous nucleation, our model offers direct applicability to nuclei-assisted cavitation in bulk liquids. We validate the new formulation by carrying out molecular dynamics simulations for nuclei a few nanometers in size (Section 3). In Section 4, we further analyze deviations between Van der Waals and ideal gas predictions. In Section 5, we compare cavitation inception pressures from our CNT with curvature and real-gas corrections, from the Blake threshold, and from MD simulations across different temperatures and nucleus sizes to assess their accuracy. Finally, Section 6 presents the conclusions.

## 2 Cavitation at gaseous nuclei – Theoretical model

Numerous models have been developed to describe cavitation. The classical model, dating back to Volmer and Weber [59], focuses on cavitation in homogeneous solutions from a thermodynamic perspective. Extensive reviews on CNT are available in the books by, Skripov [60], and Carey [61], as well as in articles by Blake [62] and Bernath [63]. Harvey et al. [27] introduced the concept of pre-existing gas cavities on surfaces as potential cavitation nuclei, stabilized by surface geometry and contact angles. Additional models consider micrometer-sized, skin-stabilized gas bubbles in liquid [64] or attached to solid surfaces [65]. While these models provide valuable insights, they fall short in explaining the high tensile strength observed in highly purified, degassed water, which experimental measurements exceed compared to theoretical predictions. This discrepancy has led to speculation that it may be due to nanoscale nuclei, such as suspended nanoscale gas bubbles or droplets [28], which are challenging to eliminate.

In this study, nanoscale gaseous nuclei are modeled with specific attention to their physical properties, rather than assuming them as simple equivalent voids. Unlike a pure void, a gaseous nucleus exhibits distinct characteristics such as internal gas pressure, surface tension, and molecular interactions with the surrounding liquid. These factors significantly impact the cavitation process, especially under high metastability conditions. Our present formulation incorporates the actual gas content and nanoscale effects, which provide a more accurate representation of cavitation behavior. The nuclei are assumed to be uniformly distributed in the liquid with an initial radius $r_{0}$​, and the surrounding liquid is treated as far from any container walls to avoid boundary effects. Under these conditions, cavitation is expected to initiate at the gaseous nuclei, with this refined model aiming to capture the influence of the gaseous nature of the nuclei on cavitation inception. The following analysis derives a mathematical model to predict cavitation pressure variations based on the properties of these nuclei and the liquid.

**Fig. 1:**
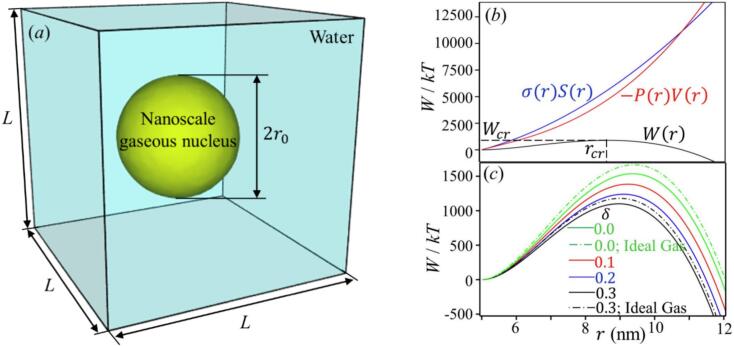
(*a*) A spherical nanoscale gaseous nucleus suspended in liquid. (*b*) Energy barrier for forming of a bubble of radius *r* from a nanoscale gaseous nucleus (black line). The blue and red lines are the first and second terms in Eq. (3), here, $r_{0}=5$ nm, $P_{l}=-15.00$ MPa, and T=298 K. (c) This plot illustrates the work required to expand the gas pocket of $r_{0}=5$ nm, for different values of Tolman length *δ*. The difference in calculated *W* between the Van Der Waals and ideal gas models is substantial. These curves reflect the balance between the free energy cost of increasing the liquid–gas interface, which dominates for smaller bubbles, and the mechanical work gained from expansion under tension, favoring larger bubbles. The peak of each curve represents the critical radius, where the free energy barrier is at its maximum.

In the framework of CNT, cavitation is an activated process in which a free energy barrier must be surpassed to form a critical nucleus, after which the bubble can grow spontaneously. The work W required to form a bubble with radius r from a nanoscale gas pocket with radius $r_{0}$ (Fig. 1a) is expressed as follows:

(1) $$W\left(r,P_{g}\right)=4\pi \left(r^{2}-r_{0}^{2}\right)\left(1-\frac{2\delta }{r}\right)\sigma_{0}+\frac{4\pi }{3}\left(r^{3}-r_{0}^{3}\right)\left(P_{l}-P_{g}-P_{v}\right),$$

where $\sigma_{0}$ is the surface tension of the planar interface, $P_{l}$, $P_{g}$, and $P_{v}$ represent the liquid, non-condensable gas, and vapor pressures, respectively. Equation (1) incorporates the curvature dependence of the surface tension through a Tolman-like correction, where Tolman length δ accounts for changes in surface tension as interface curvature increases, a key factor in nanoscale bubbles [21]. The first term in the RHS of (1) represents the energy cost required to expand the liquid–gas interface area, while the second term corresponds to the energy gained from the increase in the bubble volume. In this study, the vapor pressure taken to be the saturated vapor pressure $P_{v}^{s}$ [66]. To estimate the pressure inside the gas pocket, we employ the Van der Waals equation to account for non-ideal gas behavior at the nano-scale, which incorporates the effects of intermolecular attractions and the finite volume of gas molecules that are critical in small gas clusters. These corrections ensure more accurate modeling under conditions of high pressure and small volumes, where the assumptions of the ideal gas law no longer hold. The Van der Waals equation is expressed as follows:

(2) $$P_{g}=\frac{\mathrm{nRT}}{\frac{4}{3}\pi r^{3}-nb}-\frac{9n^{2}a}{16\pi^{2}r^{6}}$$

where n is the moles of gas in the bubble, R the universal gas constant, T the temperature, a and b are the van der Waals constants for intermolecular attraction and finite molecular volume, respectively. Combining (1) and (2), the energy barrier that must be overcome for its formation is expressed as

(3) $$W\left(r\right)=\sigma \left(r\right)S\left(r\right)+P\left(r\right)V\left(r\right)$$

where

$$\sigma \left(r\right)=\left(1-\frac{2\delta }{r}\right)\sigma_{0}$$

$$S\left(r\right)=4\pi \left(r^{2}-r_{0}^{2}\right)$$

$P\left(r\right)=P_{l}-\frac{\mathrm{nRT}}{\frac{4}{3}\pi r^{3}-nb}+\frac{9n^{2}a}{16\pi^{2}r^{6}}-P_{v}^{s}$, and

$$V\left(r\right)=\frac{4\pi }{3}\left(r^{3}-r_{0}^{3}\right)$$

Fig. 1(b) illustrates the contributions of the two terms of equation (3), $\sigma \left(r\right)S\left(r\right)$ (blue line) and $-P\left(r\right)V\left(r\right)$ (red line), to the total work $W\left(r\right)$ (black line) as functions of bubble radius r. The surface energy term, $\sigma \left(r\right)S\left(r\right)$, increases with r due to the growing surface area. The volume term, $-P\left(r\right)V\left(r\right)$, initially increasing the total work but ultimately counterbalancing the surface energy at larger radii. This interplay results in an energy barrier that rises initially, reaches a maximum $W_{\mathrm{cr}}$ at the critical nucleus radius $r_{\mathrm{cr}}$, and then decreases, allowing spontaneous bubble growth. In Fig. 1(c), the work required to expand a gas pocket is shown for various Tolman lengths (δ) [67]. The plot also highlights the difference between the van der Waals model (solid lines) and the ideal gas model (dashed-dotted lines) in predicting the work necessary for bubble expansion. As the Tolman length δ increases, both the critical work required to overcome the energy barrier and the critical radius decrease. This emphasizes the importance of incorporating curvature-dependent surface tension in nanoscale cavitation models. In this work, we select δ=0.3 Å [57,67]. The relationship between the critical radius and the liquid pressure can be derived through $\frac{\mathrm{dW}(r)}{\mathrm{dr}}=0$. After some simplification, we obtain the following equation:

(4) $$P_{l}=P_{v}^{s}-2\frac{\sigma_{0}}{r_{\mathrm{cr}}}\left(1-\frac{\delta }{r_{\mathrm{cr}}}\left(1+\frac{r_{0}^{2}}{r_{\mathrm{cr}}^{2}}\right)\right)+\left(-\frac{4nRT\pi r_{\mathrm{cr}}^{3}}{{3\left(\frac{4\pi r_{\mathrm{cr}}^{3}}{3}-nb\right)}^{2}}+\frac{9n^{2}a}{8\pi^{2}r_{\mathrm{cr}}^{6}}\right)\left(1-\frac{r_{0}^{3}}{r_{\mathrm{cr}}^{3}}\right)+\frac{\mathrm{nRT}}{\frac{4\pi r_{\mathrm{cr}}^{3}}{3}-nb}-\frac{9n^{2}a}{16\pi^{2}r_{\mathrm{cr}}^{3}}$$

**Fig. 2:**
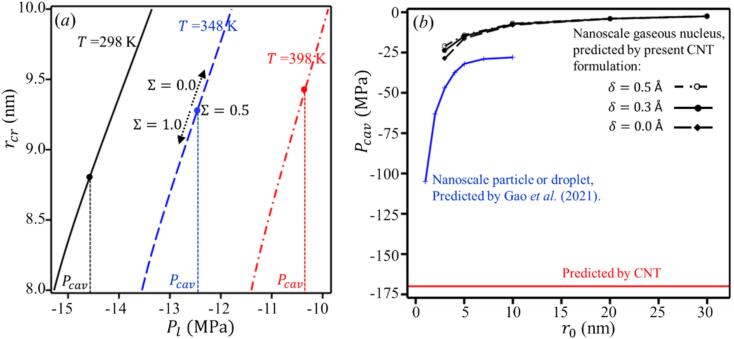
(*a*) This curve represents equation (4), illustrating the relationship between cavitation probability Σ, liquid pressure $P_{l}$, temperature and the normalized critical radius, for $r_{0}=5nm$. The cavitation pressure $P_{\mathrm{cav}}$ is defined as the liquid pressure at which Σ=0.5. As temperature increases, the magnitude of the critical pressure required for cavitation decreases. (*b*) Comparison of water cavitation pressures as predicted by the present CNT formulation (δ=0,0.3, and 0.5 Å), Gao's model, and plain CNT. This plot also demonstrates the influence of nanoscale void size on cavitation pressure.

Equation (4) involves the critical radius $r_{\mathrm{cr}}$ and the liquid pressure $P_{l}$ (Fig. 2a). By substituting the expression for $r_{\mathrm{cr}}$ in (3), the energy barrier of a critical nucleus can be expressed as follows:

(5) $$W_{\mathrm{cr}}=\sigma \left(r_{\mathrm{cr}}\right)S\left(r_{\mathrm{cr}}\right)+P\left(r_{\mathrm{cr}}\right)V\left(r_{\mathrm{cr}}\right)$$

and the equation predicting the rate of cavitation at nanoscale nuclei J, can be expressed as

(6) $$J=J_{0}\;exp\left[-\frac{W_{\mathrm{cr}}}{\mathrm{kT}}\right]$$

where $J_{0}=4\sqrt{2\sigma /\pi m}r_{0}^{2}N_{0}^{2/3}/V$, $N_{0}$ is the molecular number density of the liquid and V is the volume of the object in Fig. 1(a). The detailed derivation of $J_{0}$ is provided in Appendix A. Assuming that the liquid pressure is maintained at $P_{l}$ and the tensile stress is applied for a time Δt, the probability of cavitation Σ is given by (Fig. 2a)

(7) Σ=1-exp(-JVΔt)

In this study, we adopt the definition of cavitation pressure *P_cav_* as outlined by Caupin et al. [68] where *P_cav_* is the liquid pressure $P_{l}$ at which Σ = 1/2. Then, by applying equations (5), (6), the value of $r_{\mathrm{cr}}$ is determined. This leaves $P_{l}$ as the only unknown in equation (4), representing our *P_cav_*. Fig. 2(a) shows, as the temperature increases, the liquid pressure required for cavitation decreases, and the critical radius $r_{\mathrm{cr}}$ becomes larger. The vertical lines indicate the specific cavitation pressures $P_{\mathrm{cav}}$ at each temperature, corresponding to the point where Σ = 1/2.

As seen, the probability of cavitation increases as the $P_{l}$ decreases. Recall that in CNT, the tensile strength of a liquid depends on both the waiting time Δ*t* and the liquid volume *V* under tension [2]. From equations (6), (7), the relation between *V* Δ*t* and *Pcav* shows that *Pcav* is only weakly affected by *V* Δ*t* as it appears in a logarithmic term. For example, Zheng et al. [6] demonstrated that the predicted tensile strength changes by less than 5 % when Δ*t* varies between 0*.*001 *s* and 1000 *s*, with *V* held constant. For an ideal gas (*a* = *b* = 0), the following equation is obtained:

(8) $$P_{l}=P_{v}^{s}-2\frac{\sigma_{0}}{r_{\mathrm{cr}}}\left(1-\frac{\delta }{r_{\mathrm{cr}}}\left(1+\frac{r_{0}^{2}}{r_{\mathrm{cr}}^{2}}\right)\right)+\frac{3nRT}{4\pi r_{\mathrm{cr}}^{3}}\frac{r_{0}^{3}}{r_{\mathrm{cr}}^{3}}$$

which for uncorrected surface tension δ=0, it is reduced to:

(9) $$P_{l}=P_{v}^{s}-2\frac{\sigma_{0}}{r_{\mathrm{cr}}}+\frac{3nRT}{4\pi r_{\mathrm{cr}}^{3}}\frac{r_{0}^{3}}{r_{\mathrm{cr}}^{3}}$$

Following Herbert *et al.*^1^ and Gao *et al.*^2^, we calculate the cavitation pressure with parameters V = 2.100 × 10^−4^ mm^3^, and Δt = 4.500 × 10^−8^ s. In this study, oxygen (O_2_) is used as the non-condensable gas, with the Van der Waals constants *a* = 1*.*382 *×* 10*^−^*^1^ m^6^ *·* Pa*/*mol^2^, *b* = 3*.*186 *×* 10*^−^*^5^ m^3^*/*mol.

In Fig. 2(b), the cavitation pressures for gaseous nuclei predicted by the present CNT formulation are shown for different Tolman lengths (*δ* = 0.0, 0.3, and 0.5 Å) and compared with those for nanoscale particles or droplets as predicted by Gao et al. [52] model, together with the plain CNT prediction. The present formulation consistently yields higher cavitation pressures for gaseous nuclei, whereas modeling a nanoscale particle or droplet results in lower pressures. The CNT prediction, shown as a solid red line, represents an idealized case without nanoscale effects, leading to a much lower cavitation pressure. This comparison highlights both the effect of the Tolman correction and the importance of explicitly accounting for gaseous content in cavitation modeling, as they strongly influence the predicted inception pressures.

## 3 Comparison with molecular dynamics (MD) simulations

To validate the present CNT formulation at smaller scales, we compare its predictions with MD simulations for nuclei with radii of 3–5 *nm*. The MD solver GROMACS [69] is used with the TIP4P/2005 water molecular model [70], known for accurately replicating water properties, including surface tension [71]. Simulations are conducted in a 30 *×* 30 *×* 15 *nm*^3^ water box containing 600,000 water molecules, with 3D periodic boundary conditions. Temperature was controlled using a Nose–Hoover thermostat, and pressure was maintained with a Parrinello–Rahman barostat. The system was equilibrated under an NPT ensemble at 0.1000 MPa and 298 K for 1 ns. The gaseous nucleus was assumed to consists only oxygen (O2) molecules, and their number is calculated using (2) (Van der Waals law). In the equilibrium condition, considering the Laplace pressure across the bubble interface, we obtain the following expression:

(10) $$P_{l,0}+\frac{2}{r_{0}}\left(1-\frac{2\delta }{r_{0}}\right)\sigma_{0}=\frac{\mathrm{nRT}}{\frac{4}{3}\pi r_{0}^{3}-nb}-\frac{9n^{2}a}{16\pi^{2}r_{0}^{6}}$$

where $P_{l,0}=0.1000MPa$. In the above equation, the only unknown is number of the gas moles n (Table 1). These *n* molecules of O2 are then added to the box. To form a gaseous nucleus, we applied volume-controlled stretching, reducing system pressure to −180.0 MPa and creating a void that O2 molecules rapidly fill due to a strong diffusion gradient.

**Table 1:** Number of O_2_ molecules n inside the gaseous nucleus under the equilibrium condition, for different radius $r_{0}$ and temperature..T

T [K]	298	323	348	398	448
$r_{0}=3nm$	1001	888	776	575	408
$r_{0}=5nm$	3492	2988	2539	1816	1269

**Fig. 3:**
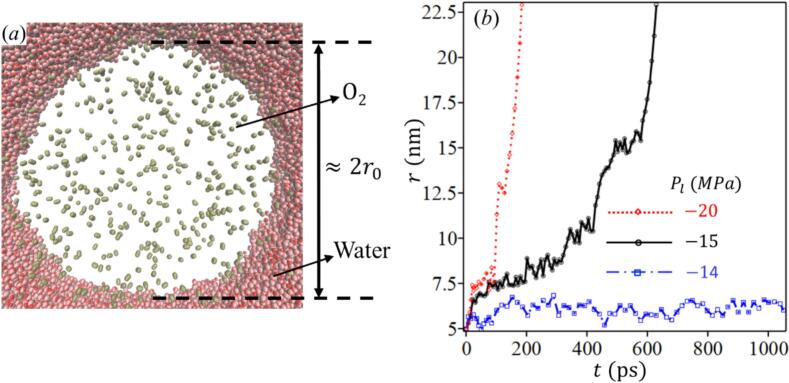
(*a*) Snapshot of the nanoscale gaseous nucleus (central cross-sectional view), here, $P_{l}$=0.1000 MPa, and T = 298 K. (*b*) Time evolution of the nanobubble radius under different $P_{l}$. The curve corresponding to $P_{l}=-14.00$ MPa (shown in blue) indicates no cavitation occurred. For lower pressures, cavitation did occur. The rate of nanobubble radius expansion increased at higher negative pressures. Here $r_{0}$≈5 nm and T = 298 K.

Within 0.5 ns, the void became densely populated, ensuring that (almost) all O2 molecules diffuse into the cavity. The pressure is then gradually adjusted to 0.1000 MPa, and the system equilibrates for 4 ns to allow the nucleus to stabilize (Fig. 3a). This method allows precise control over the nucleus diameter by adjusting the number of O2 molecules, thereby improving reproducibility in our simulations. It is important to note that once the gaseous nucleus was formed, the surrounding liquid was no longer supersaturated, as most of the O2 molecules had diffused into the void, leaving a negligible concentration of dissolved gas in the liquid phase.

### 3.1 Stability of the bulk nanobubble

Thermal capillary waves (TCWs) are thermal fluctuations occurring at gas–liquid interfaces [72], arising from a balance between molecular thermal motion and intermolecular cohesion (surface tension), while gravitational and hydrodynamic effects remain negligible at this scale (generally below 1 nm). TCWs effectively explain the reduction in surface tension [34], as confirmed by direct optical observations [73,74] and molecular dynamics simulations [75,76,77]. Reduced surface tension significantly contributes to nanobubble stability [34]. Having previously clarified phenomena such as nanodroplet coalescence [78] and nanojet rupture [79,80], TCWs represent a critical interfacial property deserving thorough investigation within the nanobubble context.

Chen et al. [34], in their recent notable publication, introduced a TCW based Epstein–Plesset model demonstrating the long-term stability of bulk nanobubbles. Their findings indicate that a bulk nanobubble with an initial radius of 2.640 nm experiences only a modest 5 % reduction in size over 100 ns, a result strongly corroborated by their direct molecular dynamics simulations. Importantly, TCWs arise naturally in molecular dynamics simulations employing the TIP4P/2005 potential, without artificial additions or modifications, as recently demonstrated explicitly by [34].

In our MD model of a nanobubble with an initial radius of *r*_0_ = 3 nm, we observed a slight reduction in bubble radius (about 4 %) within the first 10 ns. In the subsequent 90 ns, the bubble radius remained nearly constant, resulting in only about a 7 % total radius reduction over the entire 100 ns simulation. These findings align closely with the observations and model presented by [34]. While the plain Epstein–Plesset theory (without incorporating TCW effects) predicts complete dissolution of the nanobubble (having similar size of *r_0_* = 3) within a much smaller time scale, the observed stability in our MD simulations confirms the significant role that TCWs play in sustaining nanobubble longevity.

### 3.2 Cavitation inception pressure

**Fig. 4:**
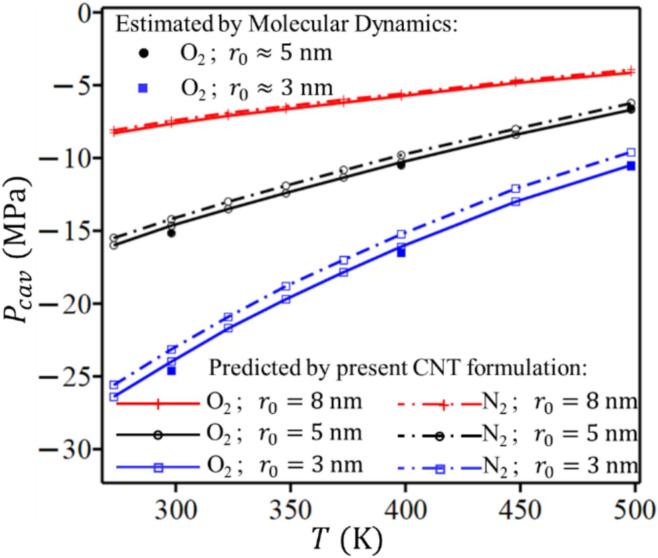
Comparison of cavitation pressure $P_{\text{cav}}$ predicted by the proposed CNT formulation and MD simulations for different nucleus sizes and temperatures. Results are shown for both O_2_ and N_2_ nuclei, with nearly identical predictions consistent with their similar van der Waals parameters.

After stabilization, the pressure is reduced once again to initiate cavitation, analyzing system’s response, now with a stable gaseous nucleus, under different negative pressures. Fig. 3b shows the dynamic behavior of the gaseous nucleus radius under varying negative pressures. Starting with a radius about 5 nm, the nucleus remained stable at −14.00 MPa, indicating internal pressure balancing external pressure. At −15.00 MPa, cavitation occurs, causing rapid expansion, which accelerated further at pressures below −20.00 MPa. Fig. 4 shows that the cavitation pressure $P_{\text{cav}}$ predicted by the proposed model agrees well with MD results for nuclei of 3 and 5 nm over a range of temperatures. The tensile strength decreases with increasing nucleus size (3–8 nm) and rising temperature (298–500 K). For $r_{0}=8$ nm, only model predictions are shown, continuing the same trend. Predictions for both O_2_ and N_2_ are included, and the results confirm that the two gases yield nearly identical cavitation pressures, consistent with their similar Van der Waals parameters. This demonstrates that the framework is applicable to different dissolved gases.

It should be noted that the present MD validation is subject to several inherent limitations. First, accessible system sizes restrict the nuclei radius to about 2–5 nm, whereas experimental cavitation nuclei may extend up to hundreds of nanometers. Second, the simulated timescales (tens to hundreds of nanoseconds) capture only the short-term dynamics of cavitation inception, while longer-term stability and growth lie beyond the reach of atomistic simulations. Third, although the TIP4P/2005 water model is among the most accurate available, no potential can fully capture water behavior under extreme negative pressures, introducing some uncertainty in the absolute values of cavitation pressure. Finally, the use of periodic boundary conditions and controlled cavity preparation differs from real experimental conditions, where dissolved gas concentrations and boundary effects may play a role. For these reasons, the MD comparison should be regarded as a nanoscale validation (few nanometers) of the proposed CNT formulation, while larger nuclei and longer timescales remain open to future investigation.

## 4 Deviation from ideal gas behavior

**Fig. 5:**
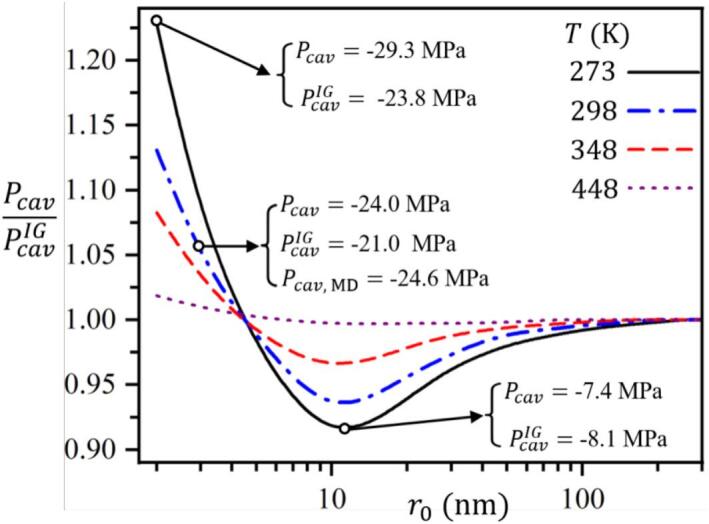
Deviation of cavitation pressure predicted by the Van der Waals model ($P_{\mathrm{cav}}$) from that predicted by the ideal gas law ($P_{\mathrm{cav}}^{\mathrm{IG}}$). Deviations are higher for smaller *r*_0_ and lower *T*. The annotations highlight specific cavitation pressures under different conditions. As temperature increases, the deviations between models decrease, indicating a reduced influence of intermolecular forces. The Tolman length correction is applied consistently to both models (*δ* = 0.3 Å).

Fig. 5 illustrates the deviation of the cavitation pressure predicted by the Van der Waals model ($P_{\mathrm{cav}}$) from that predicted by the ideal gas law ($P_{\mathrm{cav}}^{\mathrm{IG}}$). The ratio ($P_{\mathrm{cav}}/P_{\mathrm{cav}}^{\mathrm{IG}}$) shows at smaller nucleus radii, the deviation from the ideal gas prediction is more pronounced, especially at lower temperatures. This behavior is attributed to higher Laplace pressure in smaller nuclei, which amplified the effect of intermolecular forces and finite molecular size, as accounted for in the Van der Waals model. Conversely, as the nucleus radius increases, the predictions from both models converge, reflecting a diminished impact of real gas behavior. Additionally, the deviation decreases with rising temperature, as higher thermal energy reduces the influence of intermolecular forces, making the gas behave more ideally. The annotations in Fig. 5 highlight this deviation by presenting specific cavitation pressures under different conditions. For instance, at *T* = 273 K, the cavitation pressure reaches −29.30 MPa for the Van der Waals model and –23.80 MPa for the ideal gas model, while molecular dynamics simulations predict −24.60 MPa, thereby aligning more closely with the Van der Waals model.

## 5 Comparison of present CNT formulation with the Blake threshold

In this section, we present a comparative analysis of cavitation inception pressures as predicted by the Blake threshold and our new CNT formulation. The Blake threshold [81,82,83,84], provides a criterion for the stability of gas bubbles in liquids. However, we have observed that the Blake threshold may not fully capture the complexities of cavitation phenomena, especially at nanoscale. Our present CNT formulation incorporates additional factors to address these limitations, aiming to provide more accurate predictions of cavitation pressures. While previous studies have highlighted the limitations of the Blake threshold in predicting cavitation inception pressures for surface nanobubbles [85,86], there is a lack of literature addressing its accuracy in predicting cavitation inception pressure on bulk nanobubbles. We review the Blake threshold formulas for Van der Waals gases, incorporating the Tolman length correction. Assuming a constant vapor pressure $P_{v}^{s}$, the ambient liquid pressure $P_{l}$ is approximated as:

(11) $$P_{l}=P_{v}^{s}-2\frac{\sigma_{0}}{r}\left(1-\frac{2\delta }{r}\right)+\frac{\mathrm{nRT}}{\frac{4\pi r^{3}}{3}-nb}-\frac{9n^{2}a}{16\pi^{2}r^{3}}$$

The critical bubble radius, denoted as $r_{\mathrm{cr},Blake}$ can be derived through $\frac{dP_{l}}{\mathrm{dr}}=0$. Upon finding $r_{\mathrm{cr},Blake}$, cavitation inception pressure based on Blake threshold $P_{\mathrm{cav},Blake}$ will be expressed as:

(12) $$P_{\mathrm{cav},Blake}=P_{v}^{s}-2\frac{\sigma_{0}}{r_{\mathrm{cr},Blake}}\left(1-\frac{2\delta }{r_{\mathrm{cr},Blake}}\right)+\frac{\mathrm{nRT}}{\frac{4\pi r_{\mathrm{cr},Blake}^{3}}{3}-nb}-\frac{9n^{2}a}{16\pi^{2}r_{\mathrm{cr},Blake}^{3}}$$

**Fig. 6:**
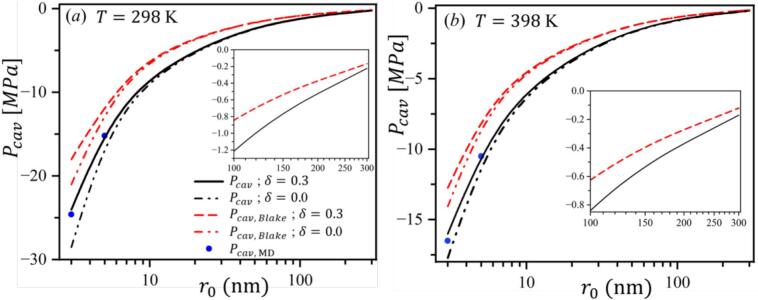
Comparison of $P_{\mathrm{cav}}$ predicted by the proposed CNT formulation, the Blake threshold and molecular dynamics simulations for different $r_{0}$, at (a) *T* = 298 K and at (b) *T* = 398 K. The present CNT formulation predicts lower cavitation pressures than the Blake threshold and aligns more closely with molecular dynamics simulations.

Although $r_{0}$ does not explicitly appear in the Eq. (12), it is inherently linked to *n* through Eq. (10). We compare cavitation inception pressures from our present CNT ($P_{\mathrm{cav}}$), from the Blake threshold ($P_{\mathrm{cav},Blake}$), and from MD simulations ($P_{\mathrm{cav},MD}$) at 298 K and 398 K, and for different Tolman lengths *δ* as a function of bubble radius, as shown in Fig. 6. Calculations show that the Blake threshold predicts higher cavitation pressures. For example, at$r_{0}$ = 5 nm and *T* = 298 K, it gives $P_{\mathrm{cav},Blake}$ = *-*11*.*60 MPa, while the present formulation predicts$P_{\mathrm{cav}}$ = *-*14*.*65 MPa, highlighting a notable discrepancy between the models. While $P_{\mathrm{cav},Blake}$ predicts higher cavitation pressures, the present CNT formulation ($P_{\mathrm{cav}}$) closely matches MD results, at least for $r_{0}$ *≤* 5 nm, where simulations were computationally feasible. This agreement indicates that our new Classical Nucleation Theory formulation provides a more accurate prediction than the Blake threshold within the studied range. The present CNT formulation (4) achieves greater accuracy by incorporating higher-order corrections compared to the Blake threshold (12), enabling a more precise representation of nanoscale effects.

## 6 Conclusions

Experimental observations indicated that the water’s tensile strength is consistently lower than theoretical estimates, even after purification and degassing, due to the unavoidable presence of nanoscale gaseous nuclei. This study presents a Classical Nucleation Theory formulation that incorporates Van der Waals corrections to account for intermolecular forces and the Tolman length to capture curvature-dependent surface tension effects. Unlike previous CNT-based approaches, which typically assumed ideal gases or constant surface tension, the present formulation combines real-gas and curvature corrections in a single framework explicitly tailored for gaseous nanonuclei, thereby providing a novel and more physically consistent description of cavitation inception. The refined framework provided a cohesive description of cavitation inception in nanoscopic scales. The present CNT formulation was validated using molecular dynamics simulations. These results highlighted the critical influence of nanoscale gaseous nuclei in reducing the water’s tensile strength, offering a comprehensive tool for evaluating their role in cavitation phenomena. We also showed that the present CNT formulation provided more accurate cavitation inception pressure predictions than the Blake threshold, thereby closely matching molecular dynamics simulation results. Furthermore, the deviations between $P_{\mathrm{cav}}$ predicted by Van der Waals and ideal gas models showed the importance of real gas behavior, particularly for small nuclei and low temperatures. For nuclei with $r_{0}<10$ nm, accounting for the Tolman correction is essential, as also illustrated in Fig. 6, whereas for larger nuclei its influence becomes negligible and the model, by setting δ=0, effectively reduces to a Van der Waals–only description.

The discrepancy between $P_{\mathrm{cav}}$ predicted by present CNT formulation and Blake threshold for bulk nanobubbles still requires further investigation through experiments or molecular dynamics simulations. A significant challenge is the lack of experimental data on cavitation inception pressure in bulk nanobubbles, as their small size makes direct observation technically difficult. Although the present study focused on O_2_ nuclei, the formulation is general and applicable to other dissolved gases. N_2_ would lead to nearly identical predictions, while gases such as CO_2_, CO, CH_4_, or NO could shift cavitation thresholds depending on their intermolecular interactions. A systematic comparison of different gases and mixtures requires further investigation and represents a promising avenue for future work. Similarly, the absence of molecular dynamics simulations addressing cavitation inception pressure in bulk nanobubbles represents a gap that future research should aim to fill.

## CRediT authorship contribution statement

**Mazyar Dawoodian:** Writing – original draft, Visualization, Validation, Software, Methodology, Investigation, Formal analysis, Conceptualization. **Ould el Moctar:** Writing – review & editing, Supervision, Methodology, Funding acquisition, Conceptualization.

## Declaration of competing interest

The authors declare the following financial interests/personal relationships which may be considered as potential competing interests: Ould el Moctar reports financial support and article publishing charges were provided by University of Duisburg-Essen Faculty of Engineering. Ould el Moctar reports a relationship with University of Duisburg-Essen Faculty of Engineering that includes: employment. If there are other authors, they declare that they have no known competing financial interests or personal relationships that could have appeared to influence the work reported in this paper.

## Appendix A . Derivation of the Prefactor$J_{0}$

The cavitation rate is expressed as

(A1) $$J=J_{0}exp\left(-\frac{W_{\text{cr}}}{k_{B}T}\right),$$

where the prefactor $J_{0}$ follows from the saddle–point approximation of the nucleation barrier. The Zeldovich factor [87,88],

(A2) $$Z=\sqrt{\frac{|W^{\prime \prime }\left(r_{\text{cr}}\right)|}{2\pi k_{B}T}},$$

accounts for the curvature of the free-energy barrier at the critical nucleus. The full expression for $W^{\prime \prime }\left(r\right)$ (derived from Eq. (3)) contains several contributions; however, for nanometer-sized nuclei the terms proportional to the gas content ($n,n^{2}$) and higher-order Tolman corrections (δ/r≪1) are negligible. Retaining only the dominant surface-tension and pressure contributions gives

(A3) $$\frac{W^{\prime \prime }}{2\pi k_{B}T}\;\;\approx \;\;\frac{4\sigma_{0}}{k_{B}T}+\frac{4r}{k_{B}T}\left(P_{l}-P_{v}^{s}\right),$$

which at the critical radius $P_{v}^{\;s}-P_{l}=\frac{4\sigma }{r_{\mathrm{cr}}}$ [2], and combining A2 and A3 one can obtain

(A4) $$Z\approx \sqrt{\frac{4\sigma_{0}}{k_{B}T}}$$

The kinetic attachment frequency $f^{+}$, describing the rate of molecular impingement on the nucleus surface, can be written as

(A5) $$f^{+}=\sqrt{\frac{k_{B}T}{2\pi m}}\;\frac{4r_{0}^{2}}{V}N_{0}^{2/3},$$

with m the molecular mass, $r_{0}$ the initial void radius, $N_{0}$ the molecular number density, and V the system volume. Combining Z and $f^{+}$ [50] yields the compact prefactor used in this work:

(A6) $$J_{0}\;\;\approx \;\;\frac{4}{V}\;\sqrt{\frac{2\sigma }{\pi m}}\;r_{0}^{2}N_{0}^{2/3}$$
